# Impact of temperature on the temporal dynamics of microcystin in *Microcystis aeruginosa* PCC7806

**DOI:** 10.3389/fmicb.2023.1200816

**Published:** 2023-08-31

**Authors:** Souvik Roy, Arthur Guljamow, Elke Dittmann

**Affiliations:** Department of Microbiology, Institute for Biochemistry and Biology, University of Potsdam, Potsdam, Germany

**Keywords:** cyanobacteria, *Microcystis*, temperature, microcystin, RubisCO, biocondensates

## Abstract

Cyanobacterial blooms pose a serious threat to water quality and human health due to the production of the potent hepatotoxin microcystin. In microcystin-producing strains of the widespread genus *Microcystis*, the toxin is largely constitutively produced, but there are fluctuations between the cellular and extracellular pool and between free microcystin and protein-bound microcystin. Here we addressed the question of how different temperatures affect the growth and temporal dynamics of secondary metabolite production in the strain *Microcystis aeruginosa* PCC7806 and its microcystin-deficient Δ*mcyB* mutant. While the wild-type strain showed pronounced growth advantages at 20°C, 30°C, and 35°C, respectively, the Δ*mcyB* mutant was superior at 25°C. We further show that short-term incubations at 25°C–35°C result in lower amounts of freely soluble microcystin than incubations at 20°C and that microcystin congener ratios differ at the different temperatures. Subsequent assessment of the protein-bound microcystin pool by dot blot analysis and subcellular localization of microcystin using immunofluorescence microscopy showed re-localization of microcystin into the protein-bound pool combined with an enhanced condensation at the cytoplasmic membrane at temperatures above 25°C. This temperature threshold also applies to the condensate formation of the carbon-fixing enzyme RubisCO thereby likely contributing to reciprocal growth advantages of wild type and Δ*mcyB* mutant at 20°C and 25°C. We discuss these findings in the context of the environmental success of *Microcystis* at higher temperatures.

## Introduction

In the summer months, many freshwater lakes are dominated by bloom-forming cyanobacteria, which cause severe environmental and socio-economic consequences (Havens, 2008; Steffensen, 2008). Global bloom formation is expected to further rise in the Anthropocene with higher temperatures and increasing atmospheric CO_2_ concentrations (Visser et al., 2016). A particularly widespread genus is the unicellular, colony-forming cyanobacterium *Microcystis* which is infamous for the production of the potent hepatotoxin microcystin (MC) of which a large number of structurally related congeners have been described. *Microcystis* blooms commonly comprise a mixture of toxic and non-toxic genotypes that either possess or lack biosynthetic genes for the nonribosomal peptide MC (Pancrace et al., 2019). The variation in the amounts of toxin produced is mainly attributed to the varying portion of MC-producing strains in blooms during the season (Briand et al., 2009).

The impact of environmental stimuli on MC production, on the other hand, is comparatively low. MC production has been intensively investigated in *Microcystis* laboratory strains. The toxins are produced from the beginning of the logarithmic phase with variations in their production rates typically not exceeding a factor of two to three (Neilan et al., 2013). Several studies have observed a linear relationship between the MC content of cells and their specific growth rate (Orr and Jones, 1998; Long et al., 2001). MC is primarily a cell-bound toxin, but the low levels of extracellular MC show pronounced temporal variations and are particularly observed at higher cell densities (Wiedner et al., 2003; Guljamow et al., 2021). Comparison of mRNA transcript levels of the MC gene clusters revealed, among other conditions, a stimulating impact of high light, iron-limiting conditions, cell density and low temperature on MC biosynthesis (Kaebernick et al., 2000; Sevilla et al., 2008; Martin et al., 2020). The increase in *mcy* gene transcription, however, was not necessarily reflected at the metabolite level. This discrepancy was recently solved by the discovery of a protein-bound pool of MC that is not extracted by methanol and accumulates primarily under conditions with increased *mcy* transcription (Meissner et al., 2013).

The analysis of protein binding partners of MC revealed a frequent affiliation of proteins to the Calvin Benson cycle including the small and large subunit of the CO_2_-fixing enzyme RubisCO (Zilliges et al., 2011; Wei et al., 2016). This finding was particularly interesting because major differences in the inorganic carbon adaptation were observed in parallel between the wild-type and the Δ*mcy*B mutant strain, with an inverse advantage at low and high CO_2_ conditions, respectively (Jähnichen et al., 2007; Van de Waal et al., 2011). RubisCO is a bifunctional enzyme whose carboxylation activity is compromised by the alternative substrate O_2_. To promote the carboxylation reaction, cyanobacteria have evolved a carbon concentrating mechanism based on two sub-components: (1) the presence and activation of a complement of bicarbonate and CO_2_ uptake transporters and (2) the encapsulation of RubisCO in carboxysomes (Burnap et al., 2015). In the context of this mechanism, which is ubiquitous in cyanobacteria, it is extremely interesting that *Microcystis*, of all cyanobacteria, exhibits a plasticity in the content of bicarbonate uptake transporter genes (Sandrini et al., 2014). Moreover, a detailed analysis of the subcellular localization of RubisCO in *Microcystis aeruginosa* PCC7806 revealed a frequent localization of RubisCO underneath the cytoplasmic membrane rather than within carboxysomes, especially under high cell density and high light conditions that were also shown to promote MC expression and condensate formation of MC (Barchewitz et al., 2019). Localization of RubisCO at the cytoplasmic membrane was connected with a high accumulation of the RubisCO product 3-phospho-glycerate and was consistent with a growth advantage of the toxin-producing wild type (Barchewitz et al., 2019). RubisCO has a tendency for liquid–liquid phase separation (LLPS) during the formation of carboxysomes in cyanobacteria as well as pyrenoids in the green alga *Chlamydomonas reinhardtii* (Wang et al., 2019; He et al., 2020). The formation of biocondensates via LLPS may be stimulated by intrinsically unstructured proteins but also by ligands and is generally promoted at the membranes (Alberti et al., 2019). In a recent study, we could demonstrate that RubisCO condensate formation in *Microcystis* is dynamic and may be stimulated by extracellular MC (Guljamow et al., 2021). Hence, there is increasing evidence that *Microcystis* strains employ a multi-level strategy in their inorganic carbon adaptation, which is not only reflected in their genotypic and phenotypic plasticity, but also involves non-canonical regulation at the protein level, presumably dependent on MC and other secondary metabolites.

Here, we have analyzed the impact of different temperatures on MC dynamics and RubisCO condensate formation. Presence or absence of MC leads to opposing effects on growth at different temperatures ranging from 20°C to 35°C. Short-term temperature shift experiments lead to pronounced differences in the intracellular and extracellular dynamics of MC and other secondary metabolites in *M. aeruginosa* PCC7806. Our data suggest that temperature is one of the critical factors for MC and RubisCO condensate formation and thereby significantly influences dynamics of soluble MC as well as growth of *M. aeruginosa* PCC7806.

## Materials and methods

### Cultivation conditions

*Microcystis aeruginosa* PCC7806 was cultivated in BG-11 medium. Chloramphenicol in a final concentration of 5 μg mL^−1^ was added for cultivation of the Δ*mcyB* mutant (Dittmann et al., 1997). For the growth curve experiments, the cultures were diluted with fresh medium to OD_750_ of 0.1 once logarithmic growth was achieved and split into three triplicates that were analyzed in parallel throughout the experiment. Diluted triplicates of *M. aeruginosa* cultures were then cultured simultaneously in an AlgaeTron AG130 growth chamber (Photo Systems Instruments, Drasov, Czech Republic) under continuous low-light condition (16 μmol photons m^−2^ s^−1^) at temperatures ranging from 20°C to 35°C, each temperature at a time. To estimate the growth, optical density at 750 nm of each replicate was determined photometrically (Novaspec III, Amersham Biosciences, United Kingdom) once a day for 14 days. For the temperature shift experiments, cells were pre-cultured at 23°C on a benchtop under a natural day and night cycle without external aeration or agitation until cultures reached a medium cell density of 0.6 ± 0.02 OD_750_. Cells were harvested by centrifugation, washed twice with growth medium, resuspended in fresh medium and then equally distributed into three conical flasks. Cell cultures were grown in an AlgaeTron AG130 for 48 h in continuous low-light condition (16 μmol photons m^−2^ s^−1^) at 20°C with continuous agitation on an orbital shaker at 72 rpm (acclimatization period) followed by a 24-h test period during which only the temperature was modified to 20°C (control condition, no change), 25°C, 30°C or 35°C. During the test period, samples were collected on the 16th, 20th & 24th hour after the temperature shift.

To determine the maximum growth rates, slope (*m*) was calculated between optical density measurements at 750 nm, from the 9th to the 14th day. The analysis was performed for both strains, encompassing the entire range of temperatures under investigation.

To assess the potential temperature-induced effects on the morphology of *M. aeruginosa*, cell diameters were determined. Diameters of multiple cells (*n* = 18) were measured across diverse micrographs. The measurement procedure was performed by the Zeiss ZEN Lite software tool (version 3.8), and calculations were based on the assumption of a circular cellular morphology.

Cell counts were conducted to examine the correlation between cell quantities and optical density values at 750 nm, measured at a temperature of 25°C. Three biological replicates of both wild-type *M. aeruginosa* and the Δ*mcyB* mutant were prepared identically to previously performed growth curve experiments and incubated at 25°C in an AlgaeTron AG130. Culture samples were collected daily for 14 days from each replicate and subsequently, cells were counted under—microscope at 40× magnification in Neubauer’s hemocytometer chamber with 0.02 mm chamber depth. Simultaneously, cell densities were photometrically measured at 750 nm (Novaspec III, Amersham Biosciences, United Kingdom).

### Protein extraction

Cell pellets from 25 mL liquid cell culture were resuspended in 1.5 mL of native extraction buffer (50 mM HEPES; 5 mM MgCl_2_ × 6H_2_O; 25 mM CaCl_2_ × 2H_2_O; 10% glycerol; pH 7). Phenylmethylsulfonyl fluoride (PMSF) was added at a final concentration of 1 mM. Samples were disrupted using a cell disruptor (Constant Cell Disruption Systems, Constant System Ltd., England) at 40 Kpsi (2,757.9 bar). Samples obtained from the cell disruptor were first centrifuged at 2,000 × g for 3 min, 4°C to pellet unbroken cells. Supernatants were further centrifuged at 13,000 × g for 10 min, 4°C, thus separating the cytoplasmic protein fraction (supernatant) from the membrane-associated protein fraction (pellet).

### SDS-PAGE & immunoblotting

For SDS-PAGE, the Bis-Tris buffer system was used along with variable (8%–15%) polyacrylamide concentrations for gels (BiteSize Bio based on NuPAGE Invitrogen, Carlsbad, CA, United States). The protein samples were added with loading dye (5x concentrated): 250 mM Tris pH 6.8, 0.1% bromophenol blue, 50% glycerol, 10% SDS, 500 mM 2-mercaptoethanol) and dithiothreitol (DTT) at a final concentration of 200 mM. All protein samples were heated at 95°C for 10 min and centrifuged for 1 min at 13,000 × g to remove possible cell debris before loading on the gel. Protein concentrations were measured using Bradford assay and samples were later normalized to equal concentrations, resulting in equal amounts of protein loaded in each lane. The protein ladder was PageRuler Plus Pre-stained (Thermo Fisher Scientific, Waltham, MA, United States). The gel was run at a voltage of 80 V for an initial 15 min followed by a constant 160 V in a MOPS running buffer (10.46 g L^−1^ MOPS, 6.06 g L^−1^ Tris, 1 g L^−1^ SDS, 0.3 g L^−1^ EDTA). Images were taken in ChemiDoc MP Imaging System (Bio-Rad, Germany). For Native-PAGE, a similar setup was used except cells were added with Native-PAGE 5× loading dye (without SDS) and samples were not heated before loading into Bis-Tris gel. Also, Native-PAGE MOPS running buffer (not containing SDS) was used for electrophoresing the samples.

For immunoblot analysis, protein gels were blotted with a wet blot electrophoresis apparatus (Mini-Protean, Bio-Rad, Hercules, CA, United States) onto nitrocellulose membranes (Amersham Protein Premium 0.45 μm MC; GE Healthcare, Chicago, IL, United States). The transfer buffer (14.42 g L^−1^ glycine, 3.03 g L^−1^ Tris) contained 20% methanol (*v*/*v*). Membranes were blocked with 1% polyvinylpyrrolidone (PVP) K-30 in TBS-T (6.06 g L^−1^ Tris, 8.77 g L^−1^ NaCl, pH 7.4, 0.1% (*v*/*v*) Tween-20, Sigma-Aldrich, St. Louis, MO, United States) and washed once for 5 min, 4°C with TBS-T. Primary antibodies were incubated in TBS-T overnight at 4°C with the following concentrations: RbcL 1:10,000 (anti-RbcL, RubisCO large subunit, form I (affinity purified), AS03037A, Rabbit, Agrisera AB, Sweden); RbcS 1:5000; CcmK 1:5000; MC 1:5000, (anti-MC-LR MC10E7, mouse; Enzo Life Sciences, Lörrach, Germany). The antibodies against *Microcystis* RbcS and CcmK were produced in our laboratory as previously described (Barchewitz et al., 2019). Membranes were washed with TBS-T and secondary antibodies (anti-mouse-IgG HRP-conjugate for MC and anti-rabbit-IgG HRP-conjugate for all other antibodies) were applied in TBS-T and incubated for at least 1 h at 4°C. Membranes were washed 4 times for 5 min, 4°C, chemiluminescence was detected with the ChemiDoc MP Imaging System (Bio-Rad, Germany). Multiple western blot experiments from different replicate experiments were performed to obtain concurrent outcomes representing the influence of temperature on target protein within the protein pool. Images shown are representatives of such observations.

Dot-blot analysis was performed to assess the influence of temperature on protein-bound MC within *M. aeruginosa* samples. To perform dot-blot analysis, equal concentrations of 4 μL protein extracts, diluted to a concentration of 10^−1^ were directly deposited onto a nitrocellulose membrane (Amersham Protein Premium 0.45 μm MC; GE Healthcare, Chicago, IL, United States). Subsequently, membranes were treated similarly to western blots and added with 1:5000 anti-MC-LR primary antibody (MC10E7, mouse; Enzo Life Sciences, Lörrach, Germany) and a secondary anti-mouse-IgG HRP-conjugate antibody. Membranes were washed 4 times, 5 min each at 4°C and chemiluminescence was detected with the ChemiDoc MP Imaging System (Bio-Rad, Germany).

### Chemical analytics

To analyze both intracellular & extracellular peptides, cell pellets from 25 mL liquid culture and 50 mL supernatant (culture medium) were taken in separate tubes. For metabolite extraction, cell pellets were resuspended in 2 mL of 100% methanol and subsequently shaken for 5 min at 3200 rpm (Vortex Genie 2; Scientific Industries, Bohemia, NY, United States). After sonication (GM 3100, Bandelin electronic, Berlin, Germany) for 10 min at 50% amplitude, 3 s on/off pulse, the sample was centrifuged for 20 min (4,700 × g, 20 min, 4°C). Supernatant collected was transferred to a fresh tube and allowed to be dried in a vacuum concentrator (RVC 2-25 CDplus; Christ, Osterode am Harz, Germany). The dried samples were resuspended in 200 μL of equal portions (1:1) 100% Methanol:MilliQ Water, and filtered using Acrodisc 4 mm with 0.45 μm membrane (Pall Life Sciences, Port Washington, NY, United States) before 20 μL of it was loaded on the high-performance liquid chromatograph Prominence LC-20 AD (Shimadzu, Kyoto, Japan) to analyze the peptides. The extracts were separated on a Symmetry Shield RP18 Column (100 Å, 3.5 μm, 4.6 mm × 100 mm) (Shimadzu Europe, Duisburg, Germany) with a mobile phase containing 0.05% Trifluoroacetic acid along with variable concentration of acetonitrile used as the organic solvent (30, 36, 100, 100, 30, 30% at 0, 12, 13, 14, 15, 17 min respectively). The quantification of peaks and examination of the chromatograms were done with LabSolutions software package (Shimadzu, Kyoto, Japan). MC and cyanopeptolins were identified based on their retention times and using MALDI-TOF MS (Bruker, Billerica, United States). Prior to MALDI-TOF measurement, sampled HPLC peak fractions were vacuum dried and resuspended in 10 μL of 20% acetonitrile −0.05% TFA. A 0.3 μL portion of the sample solution was mixed with an equal volume of an α-cyano-4-hydroxycinnamic acid matrix (3 mg/mL in 84% acetonitrile,13% ethanol, 3% water, and 0.1% TFA), spotted, and analyzed with a Bruker Microflex LRF apparatus (λ = 337 nm nitrogen laser) (Bruker, Billerica, United States) in positive-ion reflectron mode. Data were analyzed using the mMass software tool.

MC and cyanopeptolin were quantified based on quantitative standards. MC-LR [Microcystin-LR (Analytical Standard), Product ALX-350-431-C010, Enzo Life Sciences, Inc.] and (D-Asp^3^)-MC-LR [(D-Asp^3^)microcystin-LR, Product ALX-350-173-C025, Enzo Life Sciences, Inc.]. The cyanopeptolin standard was purified and weighed in our laboratory as described previously (Barchewitz et al., 2019).

### Immunofluorescence microscopy

Five mL of *M. aeruginosa* PCC7806 culture were pelleted for 1 min at 10,000 × g and washed twice with 1 mL of PBS (8.18 g L^−1^ NaCl, 0.2 g L^−1^ KCl, 1.42 g L^−1^ Na_2_HPO_4_, 0.25 g L^−1^ KH_2_HPO_4_, pH 8.3). Cell pellets were resuspended with 1 mL of 4% formaldehyde in PBS and incubated for 30 min at room temperature for the fixation process. Fixed cells were then washed twice with PBS and resuspended in 100 μL PBS. Meanwhile, 12 mm thick microscope coverslips (R. Langenbrinck GmbH, coverslips round 12 mm) were washed with 100% ethanol using ultra-sonication cleanser (Bandelin Sonorex TK52; Ultraschall-Welt, Germany) for 30 min. Twenty μL of resuspended cells were spread evenly on a microscope coverslip, slips were air-dried and stored at −20°C for later use. For antibody hybridization, coverslips with fixed samples were equilibrated in PBS for 5 min at room temperature. Afterwards, the slips were incubated with 2 mg mL^−1^ lysozyme in PBS-TX (PBS with 0.3% (*v*/*v*) Triton X-100, Sigma-Aldrich, Darmstadt, Germany) for 30 min at room temperature and washed twice with PBS-TX for 3 min. The slips were then blocked with 1% PVP K-30 in PBS-T (PBS with 0.3% *v*/*v* Tween-20, Sigma-Aldrich, Darmstadt, Germany) for at least 1 h at 4°C followed by a double washing step with PBS-T. Primary antibody dilutions in PBS-T were as follows: RbcS 1:200 RubisCO small subunit (rabbit antiserum); CcmK 1:200 (rabbit antiserum); MC 1:250 (MC-LR MC10E7, mouse; Enzo Life Sciences, Lörrach, Germany). Post-incubation of at least 1 h at room temperature, the slides were again washed twice with PBS-T and the secondary antibodies used (depending on the primary antibodies) were as follows; Alexa Fluor 488 goat anti-rabbit (1:200), Alexa Fluor 488 goat anti-mouse (1:100) and Alexa Fluor 546 goat anti-chicken (1:200); (all from ThermoFisher Scientific. Waltham, MA, United States). Subsequently, the slides were washed twice and air-dried until most of the PBS-T evaporated. A drop of ProLong^™^ Glass Antifade Mountant (Invitrogen^™^; ThermoFisher Scientific, Germany) was added to the glass slide and the dried coverslip was placed on top. The slides were allowed to cure for 24 h in the dark and were stored at −20°C until use. Laser scanning confocal micrographs were obtained using a Zeiss LSM 780 (Carl Zeiss, Oberkochen, Germany) with a Plan-Apochromat 63×/1.40 oil immersion objective. Alexa Fluor 488 was excited at 488 nm (detection spectrum 493–556 nm), Alexa Fluor 546 (570–632 nm), and autofluorescence at 633 nm (647–721 nm). The excitation was performed simultaneously.

For enhanced visualization of MC localization, immunofluorescence pixel intensities were plotted against cell diameter by analyzing multiple cells (*n* > 8) from various immunographs using the Zeiss ZEN lite software tool (version 3.8). To better visualize the temperature-induced bio-condensates among the two strains, we used the built-in 2.5D function of Zeiss ZEN lite software. In this analysis, we plotted RbcS immunofluorescence intensities as the *z*-axis parameter, while the *y*-axis and *x*-axis corresponded to the pixel coordinates.

### Statistical analysis

Analysis of variance (ANOVA) was carried out to test for statistical significance of differences in mean values using the *aov* function of R (version 4.3.0). For the growth rate determination experiment, a two-way ANOVA was carried out to test for the interaction of the parameters “strain” (i.e., either wild type or mutant) and “temperature” (i.e., the experimental temperature conditions) on the influence of the mean growth rate. Pairwise statistical significance in mean differences was determined with the *TukeyHSD* function in R using a 95% confidence interval. Compact letter display grouping was performed with the *multcompLetters4* function of the multcompView package of R.

## Results

### Effects of temperature on growth and MC production in *Microcystis aeruginosa* PCC7806 and its Δ*mcyB* mutant

**Figure 1:**
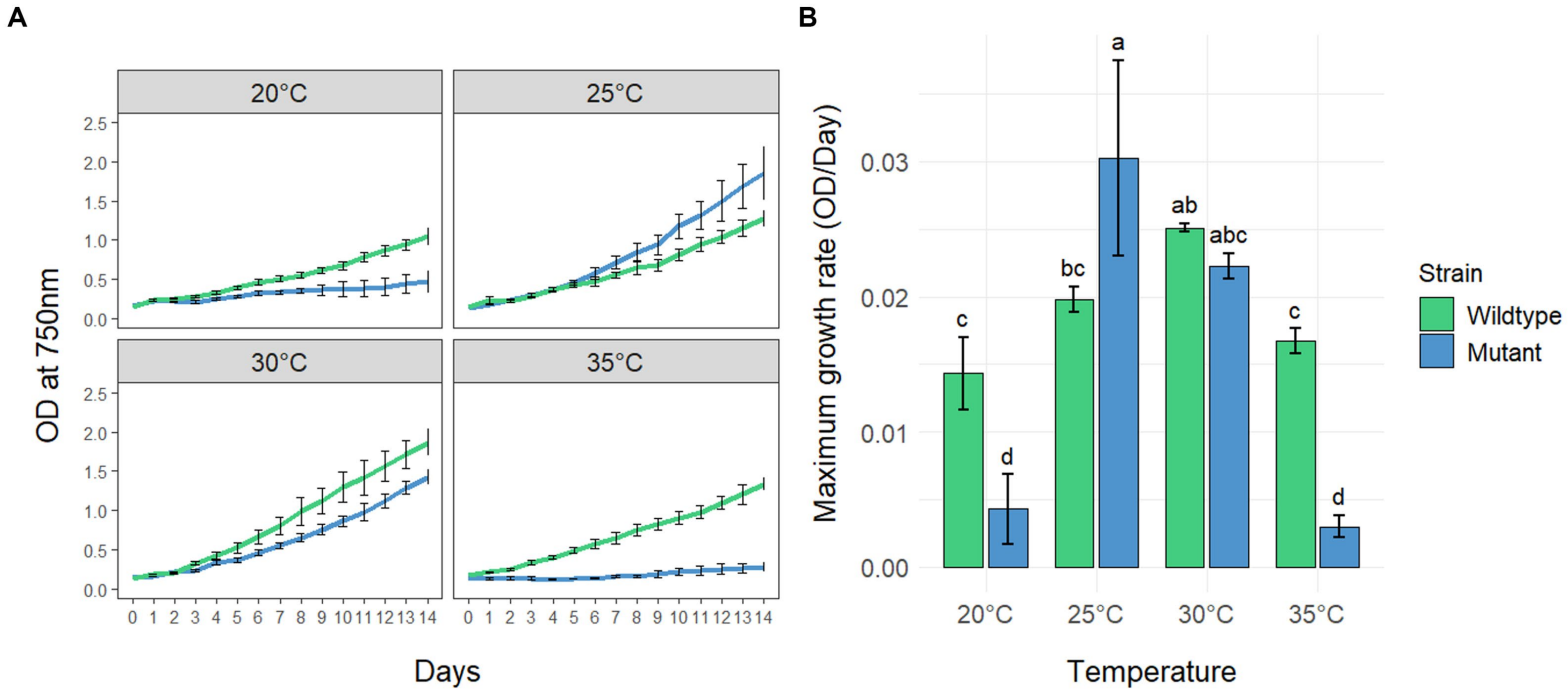
Growth of *Microcystis aeruginosa* PCC7806 (wildtype) and its MC-deficient *ΔmcyB* mutant. **(A)** Growth curves of batch cultures under continuous low light conditions. Shown are the mean OD_750_ values with standard deviations of three replicate cultures. **(B)** Maximum growth rates of the same cultures as in **(A)**. Statistically significant differences of the means across all conditions are indicated with compact letter display (CLD) after two-way ANOVA and TukeyHSD test with a 95% family-wise confidence level. CLD groups not sharing a letter differ significantly. The maximum growth rates, standard deviations and results of statistical analyses are presented in the supplementary section as Supplementary Tables S1, S2.

To investigate the effects of different temperatures on the growth of MC-producing wild-type strain PCC7806 (WT) and its Δ*mcyB* mutant, three replicates of both strains were cultivated in batch cultures for 14 days at 20°C, 25°C, 30°C and 35°C, respectively (Figure 1). Growth was monitored by measuring the optical density of the cultures at 750 nm (OD_750_, Figure 1A) as we found the OD_750_ to be a reliable and robust proxy for cell count (Supplementary Figure S1). Statistical analyses revealed that both the genetic background (WT vs. mutant) and the cultivation temperature have a significant effect on the maximum growth rate of *M. aeruginosa* (Figure 1B and Supplementary Tables S1, S2). While the Δ*mcyB* mutant strain showed maximal growth rates at 25°C, where it clearly outperformed the WT, the MC-producing strain PCC7806 grew fastest at 30°C. Moreover, the WT strain still showed robust growth at 35°C whereas the Δ*mcyB* mutant showed virtually no growth at all. Strikingly, the Δ*mcyB* mutant strain also grew poorly at 20°C where the WT strain already showed significant proliferation. Overall, growth rates of the Δ*mcyB* mutant were less robust against changes in cultivation temperature. While the growth advantages of the WT at high temperatures are in agreement with previous observations (Dziallas and Grossart, 2011), the significant growth differences at 20°C were unexpected and suggested a critical breakpoint for growth of the strain *M. aeruginosa* PCC7806 between 20°C and 25°C at the given light conditions.

**Figure 2:**
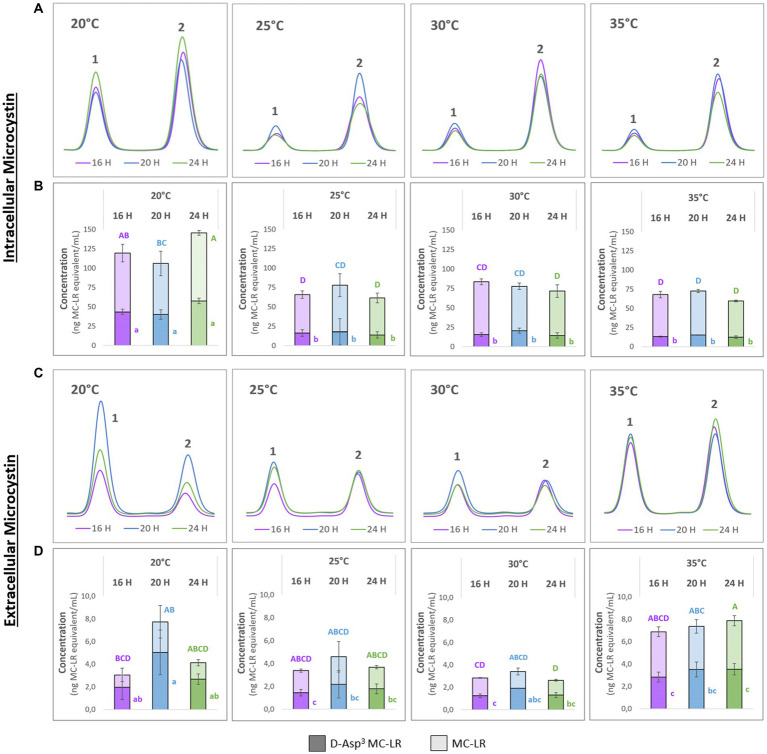
Temporal dynamics of (D-Asp^3^)-MC-LR (**1**) and MC-LR (**2**) after acclimation of precultures to 20°C and subsequent temperature shift to 20°C, 25°C, 30°C and 35°C. Samples were taken after 16, 20 and 24 h. **(A,C)** Representative HPLC profiles; **(B)** intracellular and **(D)** extracellular amounts of **1** and **2** as means with standard deviations based on three biological replicates. Statistically significant differences are indicated with compact letter display (CLD) after one-way ANOVA and TukeyHSD test with a 95% family-wise confidence level. Upper case letters are for total MC content, lower case letters are for **1** only. CLD groups not sharing a letter differ significantly.

To better understand possible temperature-dependent MC-effects we next studied the impact of temperature on MC production itself. For this purpose, we designed a temperature shift experiment where replicates of a culture of the WT strain were acclimated to 20°C, grown to medium cell density and either maintained at these conditions or exposed to 25°C, 30°C and 35°C for 24 h, respectively. To take possible dynamics of intra- and extracellular MC into account, we took samples at three different time points at 16, 20 and 24 h after the temperature shift. MC was quantified by HPLC based on a quantitative standard for MC-LR. The two major congeners produced by *M. aeruginosa* PCC7806, MC-LR and (D-Asp^3^)-MC-LR were quantified as MC-LR equivalents (Figure 2). As expected, higher amounts of MC were detected in the intracellular pool at all temperatures. While the cell-bound amount of MC was similar at 25°C, 30°C and 35°C, it was almost twice as high in cultures kept at 20°C (Figures 2A,B). Moreover, the proportion of the two congeners differed at 20°C and 25°C–35°C, respectively. While the proportion of (D-Asp^3^)-MC-LR was fluctuating around 20% at the three higher temperatures, it made up to 39% of the total intracellular MC pool at 20°C (Figures 2A,B). The extracellular MC was also strikingly different at 20°C compared to the other three temperatures. Here, too, the proportion of the (D-Asp^3^)-MC variant with up to 66% of the total MC was higher than at the other temperatures. Temporal fluctuations of extracellular MC were also most pronounced at 20°C. It is of note, that the extracellular proportion of (D-Asp^3^)-MC-LR was generally higher at all temperatures compared to the intracellular pool. The amount of extracellular MC was relatively stable at 25°C, 30°C and 35°C, but at an overall higher level at 35°C.

To better interpret the specificity of the dynamic MC effects, we additionally quantified cyanopeptolin A at the four different temperatures using a quantitative standard. In this case, we also included extracts of the Δ*mcyB* mutant that was subjected to an identical temperature shift experiment (Supplementary Figure S2). In the wild-type strain, slightly higher intracellular values were detected for cyanopeptolin A at 20°C, but the difference between 20°C and 25°C–35°C was not as significant as it was for MC (Supplementary Figure S2A). In the Δ*mcyB* mutant, no obvious difference was observed between 20°C and 25°C–35°C. While cyanopeptolin quotas were rather similar in WT and Δ*mcyB* mutant at 20°C they deviated at 25°C–35°C where the cyanopeptolin A levels remained high in the mutant but decreased in the WT, similar to MC. Generally, fewer fluctuations were detected for cyanopeptolin A in both the wild-type strain and the Δ*mcyB* mutant compared to MC, with the exception of the 35°C samples in the mutant. The lower intracellular values at 35°C in the mutant are partly correlated with higher extracellular values, so that the intracellular dynamics can probably be attributed in part to release of cyanopeptolin A (Supplementary Figure S2B).

### Temperature has an impact on MC and RubisCO condensate formation

**Figure 3:**
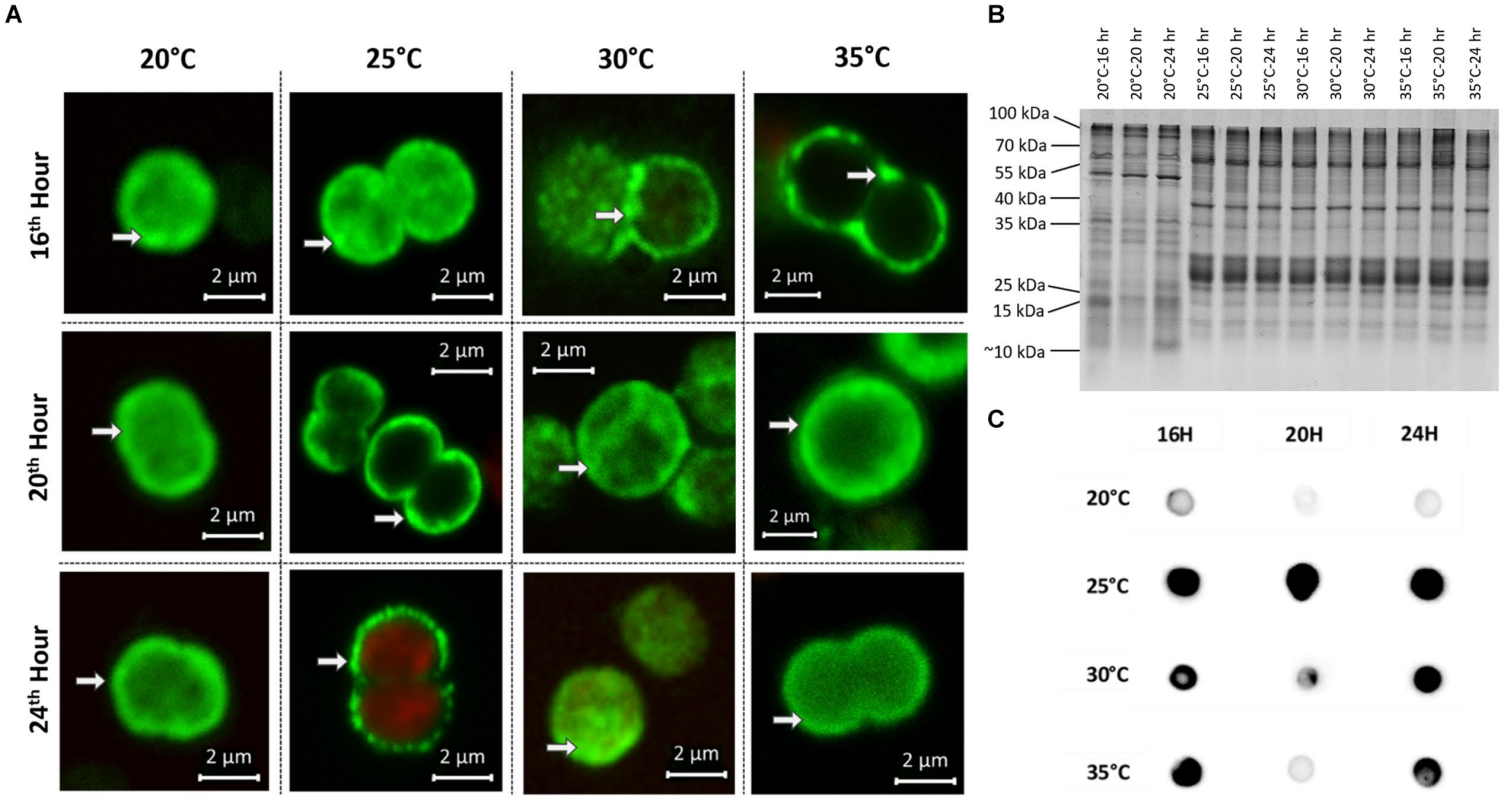
Subcellular localization and protein-binding of MC after temperature shift to 20°C, 25°C, 30°C and 35°C for 16, 20 and 24 h. **(A)** Representative MC immunofluorescence micrographs show a plasticity in the subcellular localization of MC and increased condensation of the signals at the periphery of cells at 25°C–35°C. White arrows point to peripheral MC signals. MC signals are depicted in green and autofluorescence is shown in red. **(B)** SDS-PAGE analysis of soluble protein samples displays distinct protein pattern at 20°C and 25°C–35°C. **(C)** MC immuno-dot-blot analysis shows a lower level of MC-binding to proteins at 20°C.

In addition to the samples for chemical analysis, parallel samples for immunofluorescence microscopy (IFM) and protein analysis were taken at all time points at the four different temperatures. To assess the degree of MC condensate formation and the subcellular localization of protein-bound MC, samples were analyzed by immunofluorescence microscopy using an anti-MC antibody (Figure 3A). No specific signals were obtained in control experiments, either without primary antibody (for WT) or with a combination of primary and secondary antibodies (for Δ*mcyB*, Supplementary Figure S3). Averaging the fluorescence intensity profiles across a random sample of cells (Supplementary Figure S4) we found that at 20°C, MC signals were very evenly distributed throughout the cells with an occasional enrichment at the cell’s periphery, presumably at the cell membrane. In contrast, at each of the higher temperatures, the localization of the MC signals showed a clear tendency towards increased condensation at the cell membrane compared to the 20°C samples. The extent of the condensation changed dynamically between the different time points (Figure 3A). The comparison of the soluble protein profiles showed very pronounced differences between samples exposed to 20°C and those exposed to higher temperatures (Figure 3B). While protein profiles were highly similar at 25°C–35°C, soluble protein patterns looked significantly different at 20°C, even though all samples were derived from the same pre-culture and were only exposed to the gradually different temperatures for 16–24 h. Similarly, significant differences were noted between 20°C and 25° in the MC immuno-dot-blot analysis conducted to assess the overall quantity of protein-bound MC (Figure 3C). MC protein binding amounts were similar at 25°C–35°C but fundamentally different at 20°C. Here, an overall lower tendency for MC binding was observed. Protein condensate formation at 25°C–35°C was even more apparent in Native PAGE analysis where MC was predominantly detected in large aggregates not entering the gels (Supplementary Figure S5). Both MC immunofluorescence analysis and MC immunoblot analysis therefore indicated very clear differences in MC-protein binding at 20°C, both quantitatively and qualitatively. The increased tendency to form condensates at higher temperatures could also explain the marked differences in soluble intracellular MC content at 20°C and the differences in proportions for MC congeners (Figure 2).

**Figure 4:**
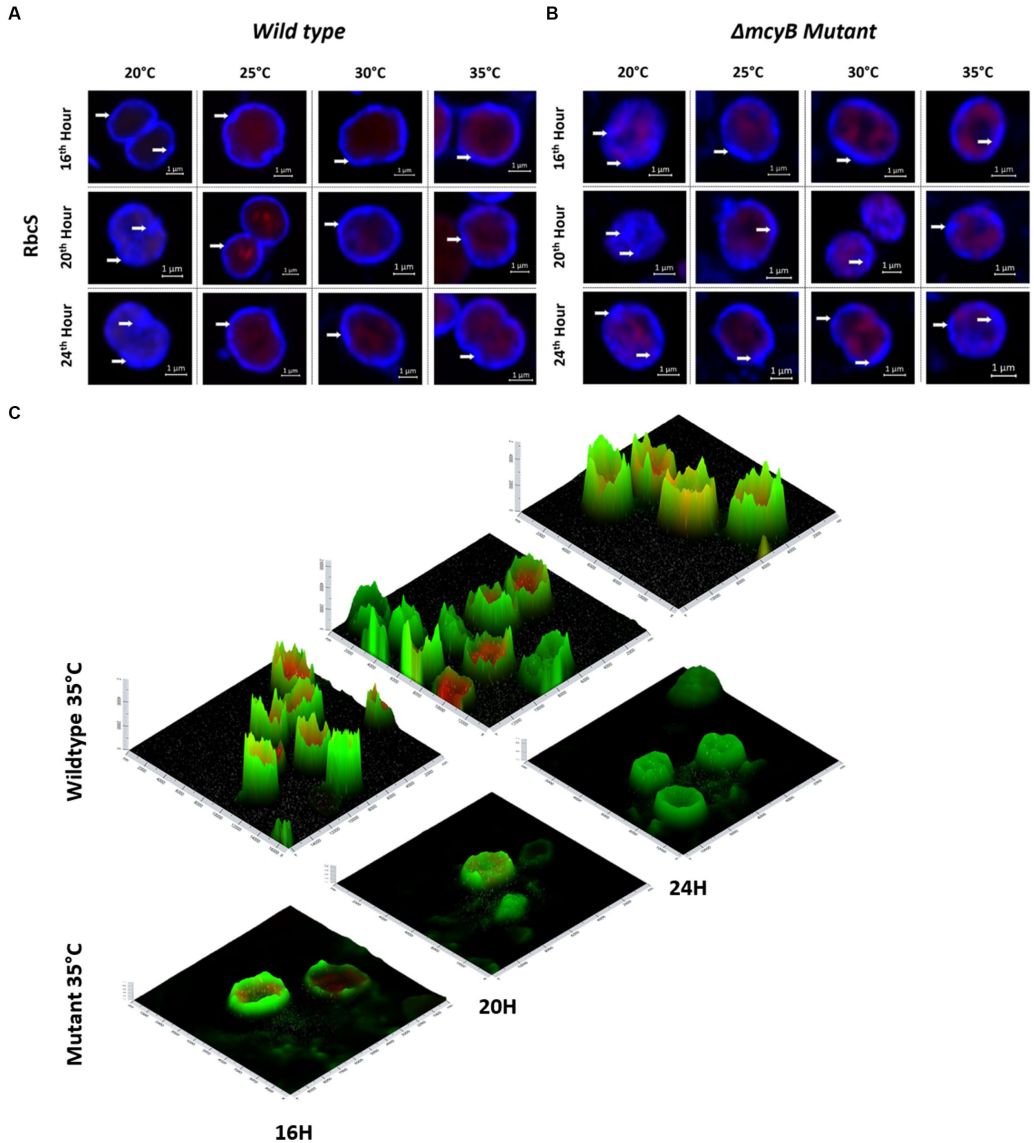
Subcellular localization of RubisCO after temperature shift to 20°C, 25°C, 30°C and 35°C for 16, 20 and 24 h. Representative RbcS immunofluorescence micrographs from **(A)** WT PCC7806 indicate a predominant cytosolic localization of RubisCO at 20°C and a membrane-associated localization at 25°C–35°C. **(B)** The Δ*mcyB* mutant displays a predominant membrane association of RubisCO at 25°C–35°C, but a lower degree of condensation than in the WT. **(C)** Spatial fluorescence intensity distribution of RbcS-derived signals at 35°C. Foci of high fluorescence intensities, appearing as “spikes” are more prevalent in the WT. RbcS signals are shown in blue **(A,B)** and green **(C)**, autofluorescence is shown in red. White arrows point to peripheral RbcS signals.

Next, we also compared the subcellular localization of the MC-binding partner RubisCO by IFM (Figure 4). In these experiments, we also included the MC-deficient Δ*mcyB* mutant. Analysis of the wild type samples revealed a predominant localization of RubisCO below the cytoplasmic membrane at 25°C–35°C. Again, the subcellular localization at 20°C was strikingly different (Figure 4A). At the lower temperature, RubisCO was predominantly evenly distributed in the cell and only occasionally appeared below the membrane. A similar trend was observed in samples of the Δ*mcyB* deficient mutant. The mutant cells, however, showed a lower degree of RubisCO condensate formation also at higher temperatures (Figure 4B; Supplementary Figure S7). At 25°C to 35°C plasticity was observed with cells either showing cytosolic localization or localization underneath the membrane. This effect is more clearly visible on intensity landscape plots of the IFM images (Figure 4C; Supplementary Figure S7). RubisCO condensates appear as local foci of fluorescence that are visualized as spikes protruding from the background level of fluorescence. Both the number and the “height” of the RubisCO-derived fluorescence foci were much lower in the Δ*mcyB* mutant, indicating an overall lower degree of RubisCO condensation (Figure 4C; Supplementary Figure S7). In agreement with this, we did not observe major differences in the soluble protein profiles of the Δ*mcyB* mutant at the four different temperatures (Supplementary Figure S6, compared with the WT, Figure 3B). To check to what extent the membrane localization also affects carboxysomal proteins, we additionally compared the localization of CcmK. Here, the typical cytosolic carboxysome structures were visible at all temperatures. Only occasionally were additional signals also visible at membranes. Wild type and mutant did not differ significantly in carboxysome localization and also showed few dynamic differences at different time points (Supplementary Figure S8). These observations indicate a clear influence of the factor temperature on the condensate formation of MC and RubisCO, but not carboxysomes. Under the selected conditions, a condensation threshold was observed between 20°C and 25°C, especially in the MC-producing WT.

## Discussion

In this study, we were able to show that production or lack of MC has a pivotal influence on the growth rate of the strain *M. aeruginosa* PCC7806 at different temperatures and that cultivation temperature, in turn, has a significant effect on the amount of freely soluble MC. We could consistently show that MC quantity, MC congener proportions, MC subcellular localization and MC binding to proteins differ markedly after short-term exposure to either 20°C or 25°C–35°C. The co-occurrence of these observations indicates that the temperature dependence and dynamics of MC depend on the condensation processes *in vivo* and that the temperature threshold for condensation is between 20°C and 25°C.

We have shown previously that MC protein condensates are not taken into account in the analysis of methanol-soluble extracts of *Microcystis* and that the amount of these condensates can change dynamically, e.g., under the influence of extracellular MC (Meissner et al., 2013; Guljamow et al., 2021). We assume that the condensation effects observed by immunofluorescence microscopy reflect the formation of MC-containing droplets by LLPS. There is mounting evidence that LLPS is a major factor in the formation of membrane-less compartments in bacterial cells and that it can have different consequences including subcellular enrichment, inactivation or activation of proteins (Alberti et al., 2019). Through protein compartmentalization, microbial cells can achieve enhanced metabolic efficiency whilst still maintaining free communication with the external environment (Gao et al., 2021). Whether or not proteins and ligands undergo phase separation depends on the nature of the proteins, their concentration and the physicochemical parameters, not least the temperature (Alberti et al., 2019). Heat-labile proteins can be protected by condensation (Gao et al., 2021). We have already described recently that MC condensation occurs almost exclusively at higher cell densities (OD_750_ > 0.5) (Wei et al., 2016; Barchewitz et al., 2019). This effect is probably triggered by higher MC and protein concentrations. Building upon these observations, the present study was designed to evaluate whether the physicochemical parameter temperature has additive or opposite effects on MC condensate formation. Indeed, we could observe that temperature has a significant influence, however, not gradually, but rather depending on a distinct threshold. In our experiment, this threshold value was between 20°C and 25°C. Given these results, the inconsistency of reports on the effects of temperature on MC production is particularly striking (Buley et al., 2022). Since many of the studies use growth temperatures between 20°C–25°C, part of the observed discrepancies may be due to the condensation processes *in vivo* and the resulting effects on MC quantifications. Stephen Wilhelm and coworkers have observed an increase in MC production after episodic temperature decrease from optimal growth temperatures at 26°C to 18°C (Martin et al., 2020). The increase in MC production was correlated with an increased MC transcription. Considering the short-term nature of the experiments tested in our study and the observed differences in MC congener proportions we believe that a lower degree of condensate formation is largely responsible for the higher amount of MC detected at 20°C. Vice versa, higher temperatures promote condensate formation leading to an apparent decrease in MC accumulation. Differences in the congener ratios may be due to differences in the propensity of different MC variants to form condensates. To be able to exactly quantify the protein-bound pool of MC would be helpful to verify this hypothesis. However, this turns out to be difficult because of the formation of large insoluble aggregates that do not enter SDS-PAGE gels and the at least partially covalent nature of MC binding to proteins (Supplementary Figure S5). We have therefore refrained from quantifying the condensates in this study and focused on demonstrating conditions favoring condensation.

While the influence of condensate formation on detectable MC quota is rather evident, the reasons for the reciprocal differences in the growth of wild-type and MC-free mutant are not well understood. Reversal of the growth advantages of the MC-producing WT strain and the Δ*mcyB* mutant has been observed repeatedly (Van de Waal et al., 2011). The mutant appears to have an advantage especially under optimal growth conditions such as elevated CO_2_ levels (Van de Waal et al., 2011). In this study, too, the mutant seems to be superior to the WT especially at optimal temperatures around 25°C while the wild type grows better under limiting growth conditions. Yet, the reasons for the growth advantage of the WT strain at 35°C and 20°C are not necessarily the same. We suspect that the differences in growth at 35°C may be due to the better stress adaptation of the wild type strain. This hypothesis is in agreement with observations of Dziallas and Grossart (2011) who compared the accumulation of stress markers in *M. aeruginosa* PCC7806 and the Δ*mcyB* mutant at higher temperatures. However, the fundamental differences in the subcellular localization of RubisCO and the global protein profiles observed between 20°C and 25°C (but *not* between 25°C and 30/35°C) could also indicate a condensation-dependent switch of metabolic states between the two temperatures. Different metabolic states could in turn explain temperature-dependent differences in growth. Metabolomic studies of *Microcystis* have shown an astounding metabolic plasticity and suggested a metabolic switch, e.g., in low-light high-light shift experiments and a possible involvement of MC (Meissner et al., 2015). Whether or not a metabolic switch occurs in temperature shift experiments has to be tested in future metabolomic studies.

Although the experiments in our study only provide a limited insight into the influence of temperature on MC production and cannot reflect the complex situation in the field, they further highlight the impact of MC on the phenotypic plasticity and environmental adaptability of *M. aeruginosa* PCC7806. *Microcystis* belongs to the cyanobacteria with a high temperature optimum of growth. Notably, the temperature optima are highly strain specific (Paerl and Huisman, 2008; Huisman et al., 2018). Our study suggests that MC and other secondary metabolites may contribute to both short and long-term adaptation of *Microcystis* to changing temperatures. Thereby, even small temperature differences can possibly lead to rapid metabolic changes. Although the condensed MC fraction is not easily quantifiable, future studies should consider temperature-dependent condensation processes when interpreting MC quota, especially in the case of sudden changes in MC amounts.

## Data availability statement

The original contributions presented in the study are included in the article/Supplementary material, further inquiries can be directed to the corresponding author.

## Author contributions

ED, AG, and SR designed research and analyzed data. SR performed experiments. SR and ED wrote the paper with contributions from all authors. All authors contributed to the article and approved the submitted version.

## Funding

This study was supported by Deutsche Forschungsgemeinschaft (DFG, German Research Foundation)—Project-ID 239748522-SFB 1127 to ED.

## Conflict of interest

The authors declare that the research was conducted in the absence of any commercial or financial relationships that could be construed as a potential conflict of interest.

## Publisher’s note

All claims expressed in this article are solely those of the authors and do not necessarily represent those of their affiliated organizations, or those of the publisher, the editors and the reviewers. Any product that may be evaluated in this article, or claim that may be made by its manufacturer, is not guaranteed or endorsed by the publisher.

## Supplementary material

The Supplementary material for this article can be found online at: https://www.frontiersin.org/articles/10.3389/fmicb.2023.1200816/full#supplementary-material
