# Supplementary material for: Impact of temperature on the temporal dynamics of microcystin in Microcystis aeruginosa PCC7806
Source: Front Microbiol. 2023 Aug 31;14:1200816. doi: 10.3389/fmicb.2023.1200816 (PMC10500830; doi:10.3389/fmicb.2023.1200816)
Supplement: Supplementary file 1 [file Data_Sheet_1.PDF]

Supplemental Information to:

Impact of temperature on the temporal dynamics of microcystin in *M. aeruginosa* PCC7806

Souvik Roy[1], Arthur Guljamow[1], Elke Dittmann[1]

[1]Department of Microbiology, Institute for Biochemistry and Biology, University of Potsdam, Karl-Liebknecht-Str. 24/25, 14476 Potsdam-Golm, Germany

**Table S1**	Two-way ANOVA test results for *M.aeruginosa* growth rates.	p.2
**Table S2**	Growth rates of *M.aeruginosa* strains at temperatures ranging from 20 to 35°C.	p.2
**Figure S1**	Evaluation of OD_{750} as a proxy for cell number.	p.3
**Figure S2**	Temporal dynamics of cyanopeptolin A after acclimation of precultures to 20°C and subsequent temperature shift to 20°C, 25°C, 30°C and 35°C, respectively.	p.4
**Figure S3**	Immunofluorescence microscopy controls for *M. aeruginosa* PCC 7806 WT and Δ*mcyB* mutant	p.5
**Figure S4**	Immunofluorescence intensity profiles of MC	p.6
**Figure S5**	Detection of MC-protein condensates at different temperatures by Native PAGE analysis	p.7
**Figure S6**	SDS-PAGE protein profiles of Δ*mcyB* mutant at different temperatures	p.8
**Figure S7**	Fluorescence intensity landscape plots of intracellular Rubisco distribution.	p.9
**Figure S8**	Subcellular localization of carboxysomes after temperature shift to 20°C, 25°C, 30°C and 35°C for 16, 20 and 24h.	p.10

Table S1. Two-way ANOVA interaction results for *M.aeruginosa* growth rates.

	Df	Sum Sq	Mean Sq	F value	Pr (>F)
**Temperature**	3	0.0013144	0.0004381	50.33	2.27e-08
**Strain**	1	0.0000980	0.0000980	11.26	0.00402
**Temperature:Strain**	3	0.0005120	0.0001707	19.61	1.31e-05
**Residuals**	16	0.0001393	0.0000087		

Table S2. Growth rates of *M.aeruginosa* strains at temperatures ranging from 20°C to 35°C.

Strain	20°C	25°C	30°C	35°C
**Mutant**	0.0043 ± 0.0026	0.0301 ± 0.0072	0.0222 ± 0.0009	0.0030 ± 0.0008
**Wildtype**	0.0143 ± 0.0026	0.0198 ± 0.0009	0.0251 ± 0.0003	0.0167 ± 0.0009

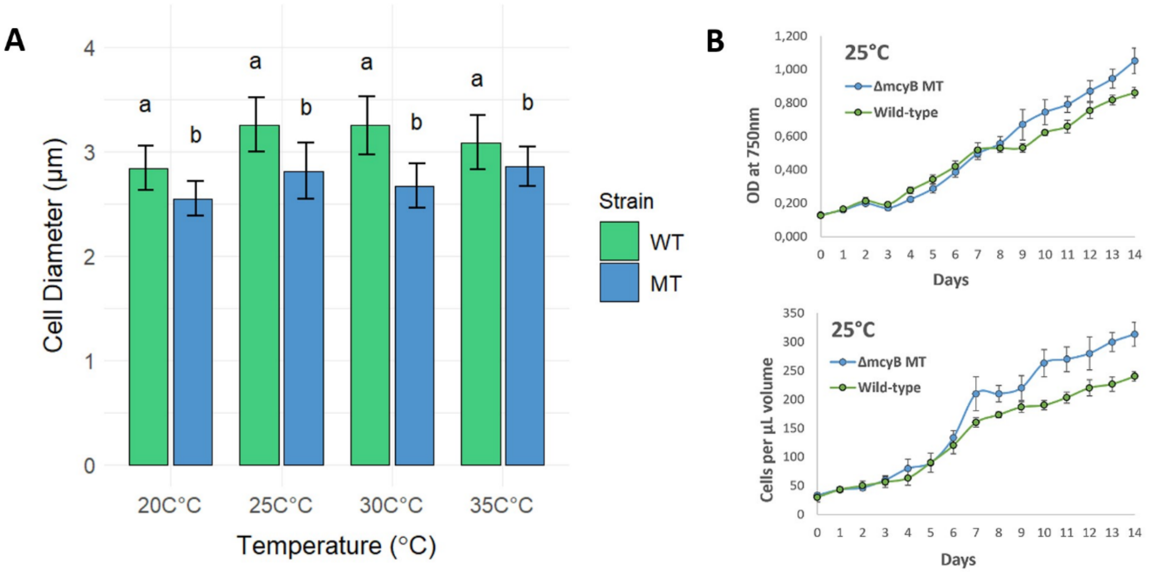

C

Strain	20°C	25°C	30°C	35°C
***mcyB*_ mutant**	2549,8 ± 166,27	2815,66 ± 268,64	2673,8 ± 211,81	2861 ± 187,01
**wildtype**	2843,66 ± 212,29	3259,06 ± 262,54	3251,53 ± 279,35	3088,66 ± 261,57

Figure S1. Evaluation of OD_{750} as a proxy for cell number.
A) Impact of temperature on cell diameter of *M. aeruginosa* strains. **B**) Comparison between growth curves measured in OD750 vs cell count. **C**) Table representing cell diameter (in nm) for *M. aeruginosa* strains across four different temperature.

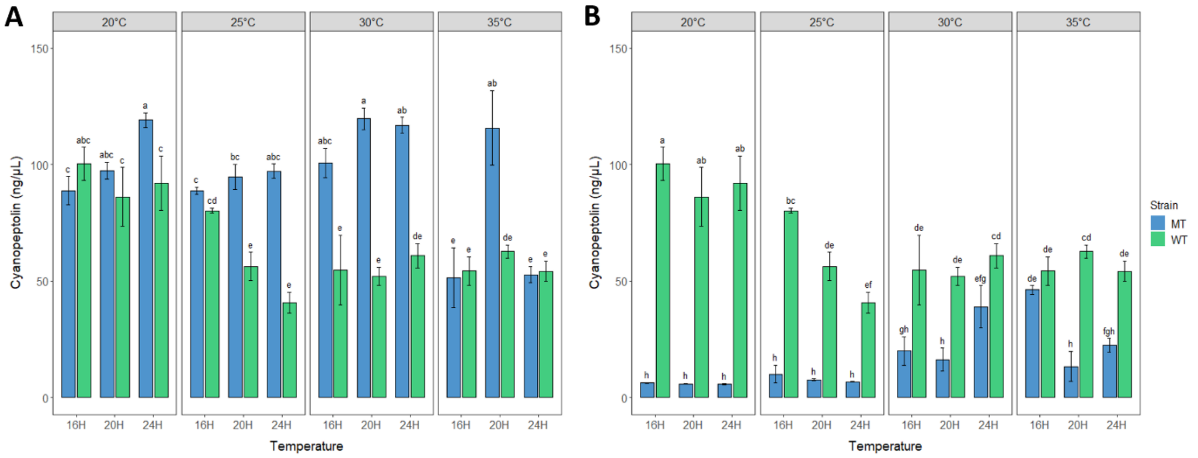

Figure S2. Temporal dynamics of cyanopeptolin A after acclimation of precultures to 20°C and subsequent temperature shift to 20°C, 25°C, 30°C and 35°C, respectively.

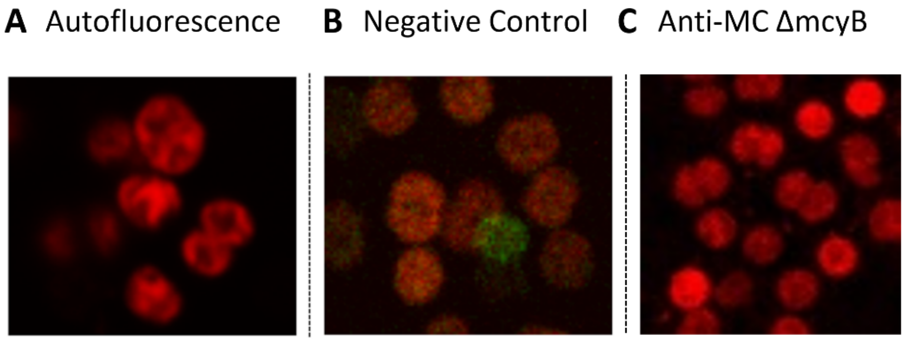

Figure S3. A) Autofluorescence of chlorophyll *a* from *Microcystis aeruginosa* PCC7806 cells without addition of fluorophore tagged antibody. **B)** Negative control from *Microcystis aeruginosa* PCC7806 wild type cells incubated with secondary antibody tagged with fluorophore to check unspecific signals. **C)** Negative control from *Microcystis aeruginosa* PCC7806 Δ*mcyB* mutant cells incubated with anti-MC primary antibody and fluorescent secondary antibody.

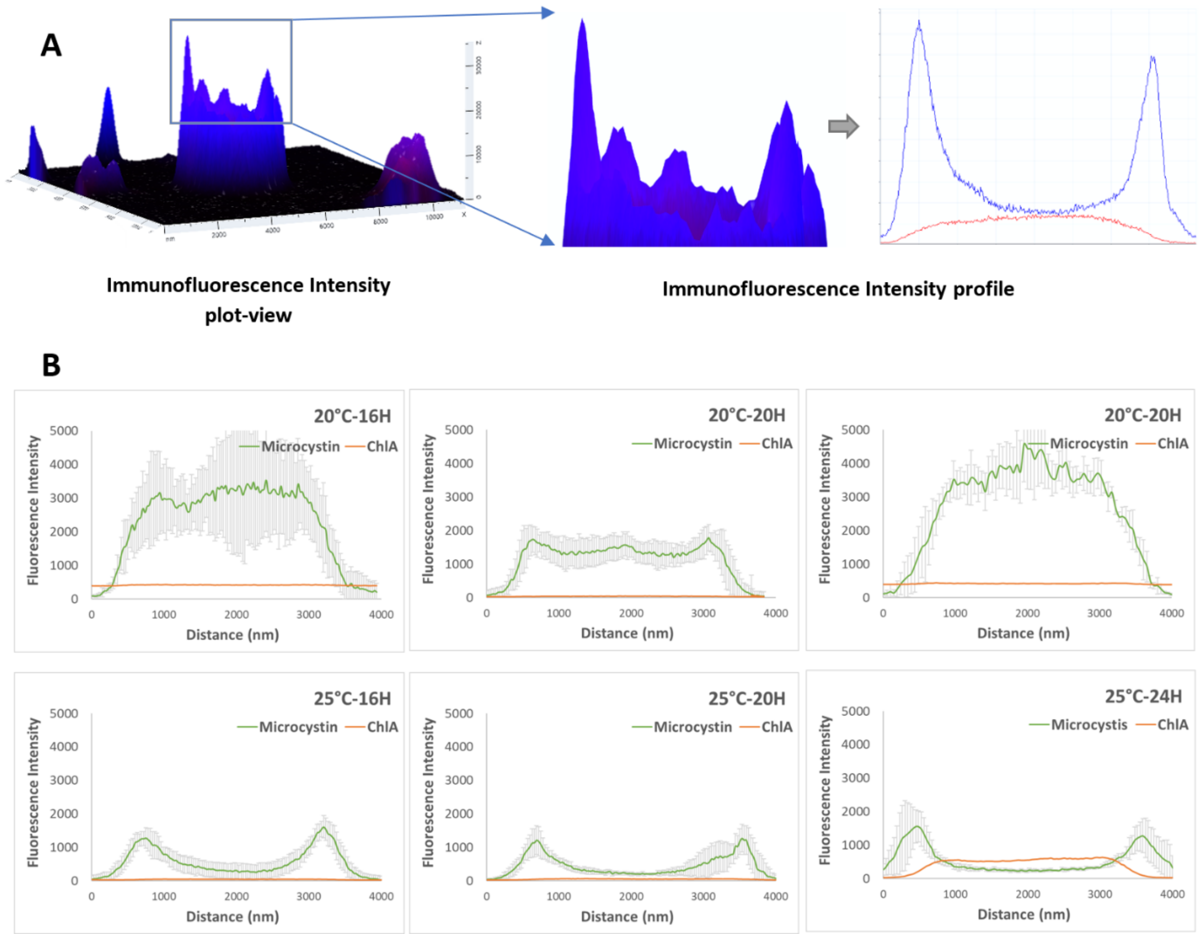

Figure S4. Immunofluorescence intensity profiles of MC.

A) Illustration for immunofluorescence intensity plot view conversion into intensity profile. **B**) Immunofluorescence intensity profiles of wildtype *M. aeruginosa* cells plotted against cell diameter (n > 8) for 20°C and 25°C, showing average localization of MC in relation to cell diameter.

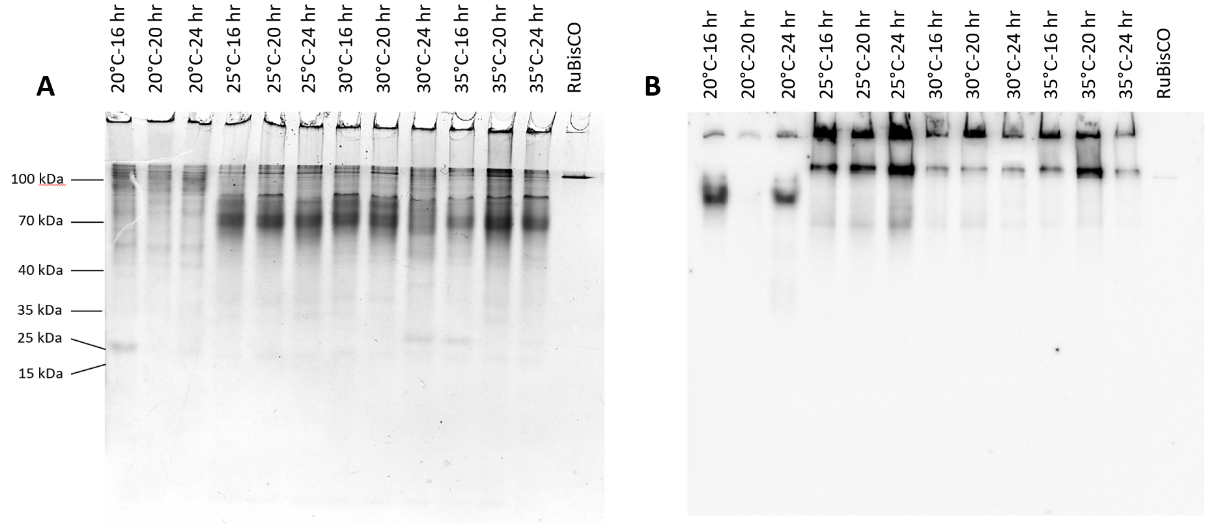

Figure S5. A) Native PAGE and B) MC immunoblot analysis of soluble protein extracts of *M. aeruginosa* PCC 7806 acclimated to 20°C and subsequently exposed to 25, 30 and 35°C, respectively shows formation of large MC-containing aggregates which do not enter the gel, especially at 25-35°C. Purified RubisCO L_8S_8 complex is included as size orientation.

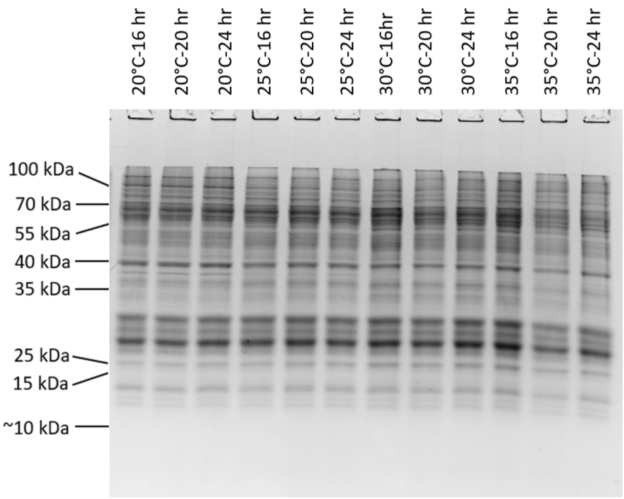

Figure S6. SDS-PAGE protein profiles of Δ*mcyB* mutant at different temperatures do not indicate major differences due to condensation effects as in the wild type (Figure 3B).

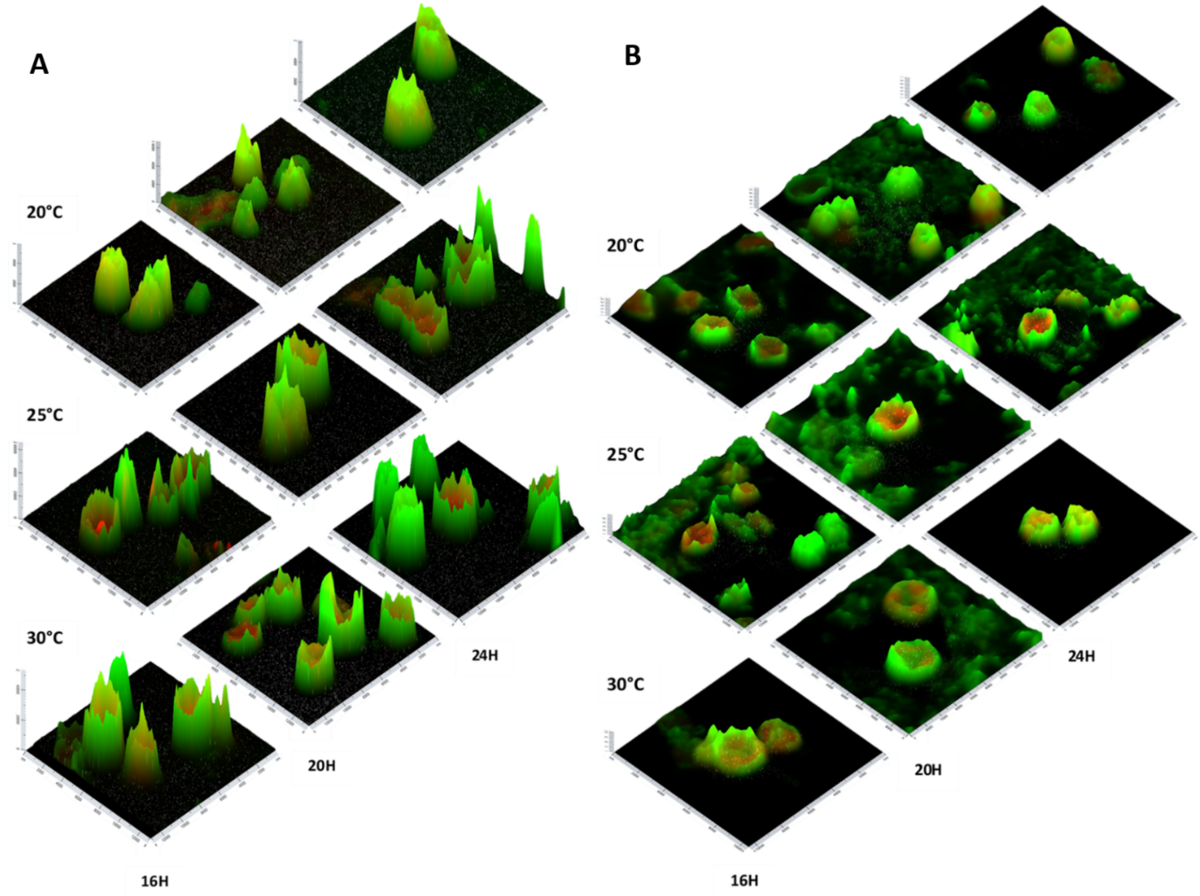

Figure S7. 2.5D fluorescence intensity landscape plots of intracellular RubisCO distribution at 20°C, 25°C and 30°C for 16, 20, 24 hours for A) WT strain *M. aeruginosa* PCC 7806 and B) the Δ*mcyB* mutant, using 2.5D plugin of Zeiss ZEN Lite software (version 3.8).

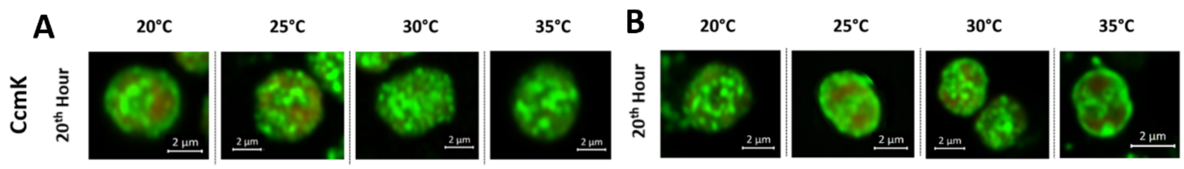

Figure S8. Subcellular localization of carboxysomes after temperature shift to 20°C, 25°C, 30°C and 35°C for 16, 20 and 24 hours. A) WT strain *M. aeruginosa* PCC 7806 and B) the Δ*mcyB* mutant
